# No Evidence for an Object Working Memory Capacity Benefit with Extended Viewing Time

**DOI:** 10.1523/ENEURO.0150-20.2020

**Published:** 2020-09-22

**Authors:** Colin Quirk, Kirsten C.S. Adam, Edward K. Vogel

**Affiliations:** 1 Department of Psychology, University of Chicago, Chicago, IL 60637; 2 Institute for Mind and Biology, University of Chicago, Chicago, IL 60637; 3 Department of Psychology, University of California San Diego, La Jolla, CA 92093; 4 Institute for Neural Computation, University of California San Diego, La Jolla, CA 92093

**Keywords:** contralateral delay activity, visual working memory

## Abstract

Visual working memory is the ability to hold visual information temporarily in mind. A key feature of working memory is its starkly limited capacity, such that only a few simple items can be remembered at once. Prior work has shown that this capacity limit cannot be circumvented by providing additional encoding time, whether providing just 200 ms or up to 1300 ms, capacity is still limited to only three to four items. In contrast, Brady et al. (2016) hypothesized that real-world objects, but not simple items used in prior research, benefit from additional encoding time and are not subject to traditional capacity limits. They supported this hypothesis with results from both behavior and the contralateral delay activity (CDA), an EEG marker of working memory storage, and concluded that familiar, complex stimuli are necessary to observe encoding time effects. Here, we conducted three replications of Brady et al.’s key manipulation with a larger number of human participants and more trials per condition. We failed to replicate their primary behavioral result (objects benefit more than colors from additional encoding time) and failed to observe an object-specific increase in the CDA. Instead, we found that encoding time benefitted both simple color items and real-world objects, in contrast to both the findings by Brady et al., and some prior work on this topic. Overall, we observed no support for the hypothesis that real-world objects have a different capacity than colored squares. We discuss the implications of our findings for theories of visual working memory (VWM).

## Significance Statement

A long-standing debate in visual working memory (VWM) has centered on the limits of working memory. VWM is thought to rely on a fixed pool of resources, but recent work by Brady et al. (2016) suggested that capacity is higher for real-world objects compared with simple stimuli. Our study attempts to replicate this result. Surprisingly, we found a performance increase for both simple and real-world stimuli at longer encoding times, but a complementary finding was not observed in the contralateral delay activity (CDA). Based on this, our data show no specific evidence for a capacity benefit for real-world items.

## Introduction

Visual working memory (VWM) is the ability to temporarily hold information in mind and is thought to be a key cognitive workspace for interfacing between perception, the contents of long-term memory, and our immediate goals. Despite this important role, the capacity of working memory is limited such that we can only hold a few pieces of information in mind at once. Although there are ongoing debates about the nature of VWM’s information limit (van den Berg et al., 2012; Adam et al., 2017), there is broad agreement that this limit is constant (i.e., a fixed pool of working memory resources are available to allocate to remembered information; Luck and Vogel, 1997; Wilken and Ma, 2004; Bays and Husain, 2008; Zhang and Luck, 2008; van den Berg et al., 2012; Adam et al., 2017). Recently Brady et al. (2016) surprisingly found that the capacity limit of working memory may actually change as a function of stimulus type and encoding time.

Before proceeding, we first need to define some key terms. In the behavioral literature, the terms “capacity” and “performance” are often used interchangeably, but in this context, they refer to distinct concepts. We will use the term performance to refer to any observed change to behavioral performance on a working memory task (e.g., changes to the behavioral measure “K”). Importantly, changes to performance can be caused by one or many underlying cognitive processes. For example, when performing a single trial of a working memory task, one needs to attend to the cued side of the display, encode the relevant stimuli, actively maintain the stimuli across a blank delay, and compare the memoranda to the test probe. A change in behavioral performance (K) could thus be because of any one or a combination of these sub-processes. In contrast, we will use the term capacity to refer to the maximum amount of information that may be actively held in working memory during the delay period. Following Brady and colleagues, capacity will be operationalized as the maximum observed amplitude of the contralateral delay activity (CDA).

Brady and colleagues found that, given sufficient encoding time, behavioral performance on a working memory task was substantially higher for familiar, complex items (i.e., images of real-world objects) than for artificial, simple items (i.e., the colored squares commonly used in prior research). In their critical behavioral experiment, Brady and colleagues examined performance as a function of encoding time (200, 1000, 2000 ms) and stimulus type (objects, colors). Regardless of display time, behavioral performance (K) for colored squares was constant at an estimated 3.5 items remembered. In contrast, performance for real-world items increased with longer encoding durations, consistent with an additional item being stored when remembering realistic stimuli at the longest encoding duration. Importantly, this behavioral result alone did not distinguish between a general performance benefit and a true increase in working memory storage capacity (i.e., a higher limit on the amount of information stored). In many ways, a general performance benefit for real-world objects would be unsurprising in light of extensive research which suggests that the capacity of long-term memory is effectively unlimited (Standing, 1973), benefits from longer encoding times (Shaffer and Shiffrin, 1972; Tversky and Sherman, 1975), and can aid in the chunking of information in working memory (Cowan, 2010). However, this performance benefit would not be diagnostic of a change to working memory storage, per se, because of the critical confounding factor of available long-term memories which may aid performance via dissociable neural mechanisms from working memory (Jeneson and Squire, 2012) and which may not be visible to a CDA analysis (Carlisle et al., 2011).

To distinguish a general performance increase from a working memory capacity increase, Brady and colleagues followed up their behavioral results with an experiment using the CDA, an event-related potential that indexes the number of items held in working memory and is highly sensitive to capacity limitations (Vogel and Machizawa, 2004). CDA amplitude increases (becomes more negative) as a function of set size and reaches a plateau at around three items. Brady and colleagues found that real-world items led to higher CDA amplitude for objects versus colors at set size 5 but not set size 3, suggesting that the object-related performance increase was because of storing more information in working memory.

The results and conclusions of the Brady et al. study present a significant challenge to nearly all extant models of visual working memory which posit that the available pool of working memory resources, as indexed by the CDA, is of fixed capacity. We therefore sought to perform a near-direct replication of the two key findings of their study.

### Overview of experiments

In our first behavioral experiment (experiment 1a), we attempted a near-direct replication of the performance benefit for real-world items described by Brady et al. Subjects completed a visual working memory task with a two-alternative forced-choice (2AFC) response as in the original paper. Surprisingly, we found increased performance with extended encoding times regardless of stimulus type, suggesting that the viewing time benefit is not exclusive to real-world items. Experiments 1b and 1c further corroborated this finding by showing the same effect with a more difficult verbal suppression task and with interleaved trials, respectively. Finally, experiment 2 attempted to reproduce the critical CDA finding showing higher amplitude in response to real-world items at set size 5, but not set size 3. We again failed to replicate the results described in the original paper, instead finding no evidence of greater amplitudes for real-world items at higher set sizes. Based on our results, we found no evidence to support the primary conclusions drawn by Brady et al. (2016). The data, experiment code, and analysis code for all experiments are available at http://osf.io/vq37u/.

## Experiment 1

### Experiment 1a

In experiment 1 from Brady et al. (2016), the authors asked participants to remember a set of six colors or real-world objects across multiple display timings to compare changes in performance across encoding times for both stimuli types. After a short delay, the participants’ memory was tested with a single 2AFC response at one of the memory locations. Foil colors were always 180° from the target color in color space, whereas foil objects were either from a different category (“objects” condition) or a different object from the same category (“objects with detail” condition). Encoding times varied across three levels: 200, 1000, and 2000 milliseconds (ms). The authors then calculated estimated working memory capacity (K) for the given stimuli and display time (Cowan, 2001). Participants simultaneously completed a verbal memory task to reduce the influence of verbal rehearsal as a memory strategy. Results from this experiment showed that K did not increase for colors at longer encoding times whereas in the objects condition, performance improved at longer encoding times. A similar pattern was observed for the objects with detail condition, although the increase was smaller.

We attempted a direct replication of the critical colors and objects conditions with all of the original display timings. In order to replicate the original experiment, we would expect to see VWM fill within 200 ms for colors, resulting in no differences across encoding times. For objects, we would expect to see an increase across the encoding times resulting in higher K at 2000-ms encoding times for objects versus colors.

#### Participants

Twelve subjects participated in experiment 1 of the original report. In order to ensure we had sufficient power to detect the effects of interest, we set an a priori sample size of 25. Volunteers (16 females, 8 males, one other/chose not to respond) aged 18–31 with self-reported normal or corrected-to-normal visual acuity and normal color vision were recruited from the University of Chicago and the surrounding area to complete the study for monetary compensation ($10/h). Participants provided their informed consent according to procedures approved by the Institutional Review Board at the University of Chicago.

#### Stimuli

Stimuli were generated in accordance with the methods reported in the original paper. Colors were selected from CIE L*a*b* color space by creating a circle with a radius of 59° centered at L = 54, a = 18, and b = −8. Sample colors were randomly chosen from 360 possible values with a minimum separation of 15° and foil colors were made to be exactly 180° away from the target value. Three thousand pictures of real-world items were used as object stimuli (http://bradylab.ucsd.edu/stimuli.html). Sample objects were randomly chosen on each trial given the requirement they all came from separate categories of items. The foil object was then randomly selected from any of the categories not included in the sample. Stimuli were displayed on an invisible ring in fixed, equidistant positions such that three stimuli were displayed in each hemifield. Stimuli were generated and displayed on a white background using MATLAB and the Psychophysics Toolbox (Kleiner et al., 2007).

#### Procedure

**Figure 1.**
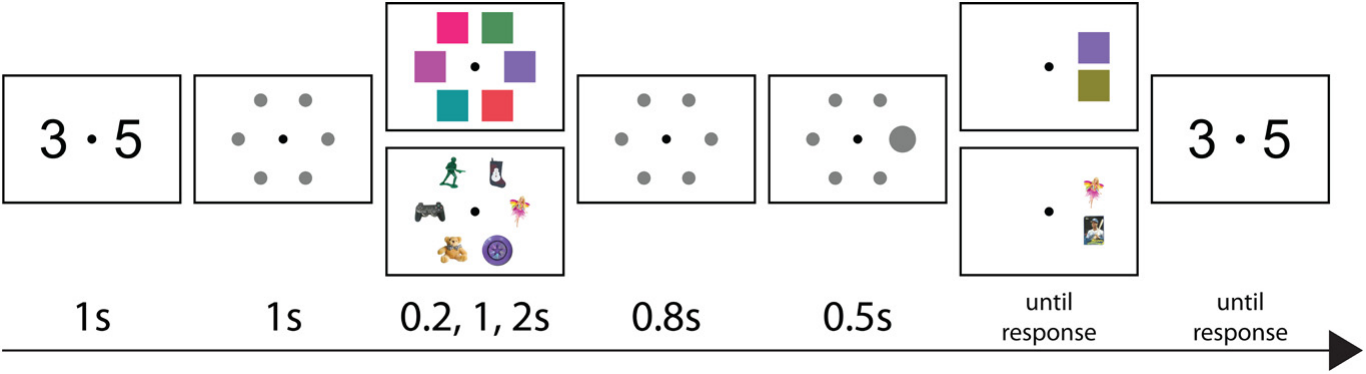
Sequence of trial events. Participants were asked to silently rehearse two digits, then six colors or objects were displayed for 0.2, 1, or 2 seconds (s). After 0.8 s, a cue appeared for 0.5 s indicating which item would be tested. The participant then responded to the 2AFC test with the arrow keys and finally recalled the remembered digits with the number keys.

Our procedure was crafted to be as close to the task reported by Brady and colleagues as possible. All displays had a fixation cross in the center of the screen and participants were instructed to avoid moving their eyes until responding. A secondary articulatory suppression task was used to prevent participants from using a verbal encoding strategy. Participants were shown two digits and asked to silently repeat them in mind while completing the working memory task. After being presented with the digits, gray placeholders showing the positions of upcoming stimuli appeared for 1000 ms. The sample display containing either six colors or six objects was then displayed for the appropriate encoding time depending on the condition block. The possible encoding times were 200, 1000, or 2000 ms. After the sample display, the placeholders reappeared for an 800-ms delay. A larger circle serving as a cue then appeared at the test location for 500 ms. After the cue, two choices were presented, one above and one below the position of the original stimulus in the test location. Participants made their response by indicating which item appeared in the original array at that location with the up and down arrow keys. Finally, participants were asked to enter the numbers from the verbal suppression task using the number keys. No time limit was placed on either response (Fig. 1).

As in Brady et al. (2016), trials were fully blocked by condition such that participants had full knowledge of the stimuli type and encoding time duration for each block of trials. All blocks were randomly ordered within the experiment for each participant. Fifty trials were displayed for each of the six condition combinations (two item types × three encoding times) giving a total of 300 trials.

#### Differences from Brady et al.(2016)

We chose to not include the objects with detail condition as we felt it was not central to the primary conclusions of the experiment. As discussed by the original authors, the objects and colors conditions are more comparable as they both focused on estimating the number of items remembered with any level of detail. This condition is also dropped in experiment 3 in the original paper. This decision allowed us to collect more trials per condition (50 vs 33 in the original experiment). To ensure we could detect the effects from the original paper, we increased the number of participants from 12 to 25.

Some details were not reported in the original paper, so reasonable values had to be selected. All stimuli were 2.1° of visual angle in size and presented in a ring 4.2° from fixation. A description of the monitor was not provided in the original text, so we cannot be confident similar equipment was used. The experiments in this paper all used 24” LCD screens with a refresh rate of 120 Hz and a resolution of 1080 × 1920. Participants were seated ∼75 cm from the screen.

#### Analysis

Our goal was not only to replicate the effect from the original paper, but to replicate the large effect sizes observed. As a result, we felt that a frequentist framework was not ideal as *p* values are uninformative for nonsignificant results and confidence intervals (CIs) cannot communicate the probability of specific effect sizes. Instead, we report Bayesian equal-tailed credible intervals to communicate our estimates and uncertainty as well as describe our level of confidence as to whether a given effect exists (Kruschke and Liddell, 2018).

Behavioral performance was calculated using the formula described in the original paper, K = N(2p – 1), where K is the estimated number of items remembered (on average for a given set size), N is the number of items to be remembered, and p is the percent of trials answered correctly for that condition. This formula is derived from *p* = 1.0 * (K/N) + 0.5 * (1 – K/N) which assumes items are either perfectly held in memory or are a complete guess. Note, the original paper contains a misprint in this formula, but the final formula used in the analyses is still correct. We further note that K is typically referred to in the literature as capacity. However, to prevent confusion or ambiguity about a general performance benefit versus a delay period-specific performance benefit, here we refer to the K measure as performance.

A hierarchical linear model was fit using performance (K) as a normally distributed response variable and encoding time and item type as interacting population level predictors. The effects of encoding time and item type were also included as group level effects and were allowed to vary over individual participants with nonzero correlation. Encoding time is treated as a categorical variable rather than as a numerical variable given the low number of factor levels and the expected nonlinearity of the time effect.

Based on our experience and the effect sizes reported in the literature, we believe that it is unlikely to observe a scaled effect >2 SDs. To formalize this belief, we used Normal(μ = 0, σ = 1) as a weakly informative, regularizing prior for all population level parameters. All group level parameters used a HalfNormal(σ = 1) prior with the exception of the intercept, which instead used HalfNormal(σ = 3) to account for the known high amount of capacity variability across individuals. For correlations among group level parameters, the default of LKJ(η **=** 1) was used as a prior for the correlation matrix. As we had no predictions for these correlations, this weakly informative prior was appropriate. Finally, the σ parameter used to model left-over variability was also given a HalfNormal(σ = 1) prior. To examine the impact of the chosen priors on our analyses, models were also fit with the less informative priors T(μ = 0, σ = 3, v = 10) and HalfT(μ = 0, σ = 3, v = 10). Posterior estimates were not noticeably different from the original analysis. One alternative possibility was to use informative priors that represented the results reported in Brady et al. (2016) in an attempt to combine the evidence from both studies. As our goal was to attempt a replication of the effects reported in the original paper as opposed to forming the best possible parameter estimates, we chose to exclude their work from informing our priors.

Parameter distributions were estimated using the No U-Turn Sampler (NUTS) implemented in Stan (Carpenter et al., 2017) and the brms package in R (Bürkner, 2017). Four Markov Chain Monte Carlo (MCMC) chains each drew 1000 warmup samples and 10,000 post warmup samples from the posterior distribution for a total of 40,000 post warmup samples. MCMC performance was assessed by confirming a value of $\hat{R}$ close or equal to 1.00 and by visual inspection of trace plots. Population level parameters each had at least 10,000 bulk and tail effective samples.

#### Results

**Figure 2.**
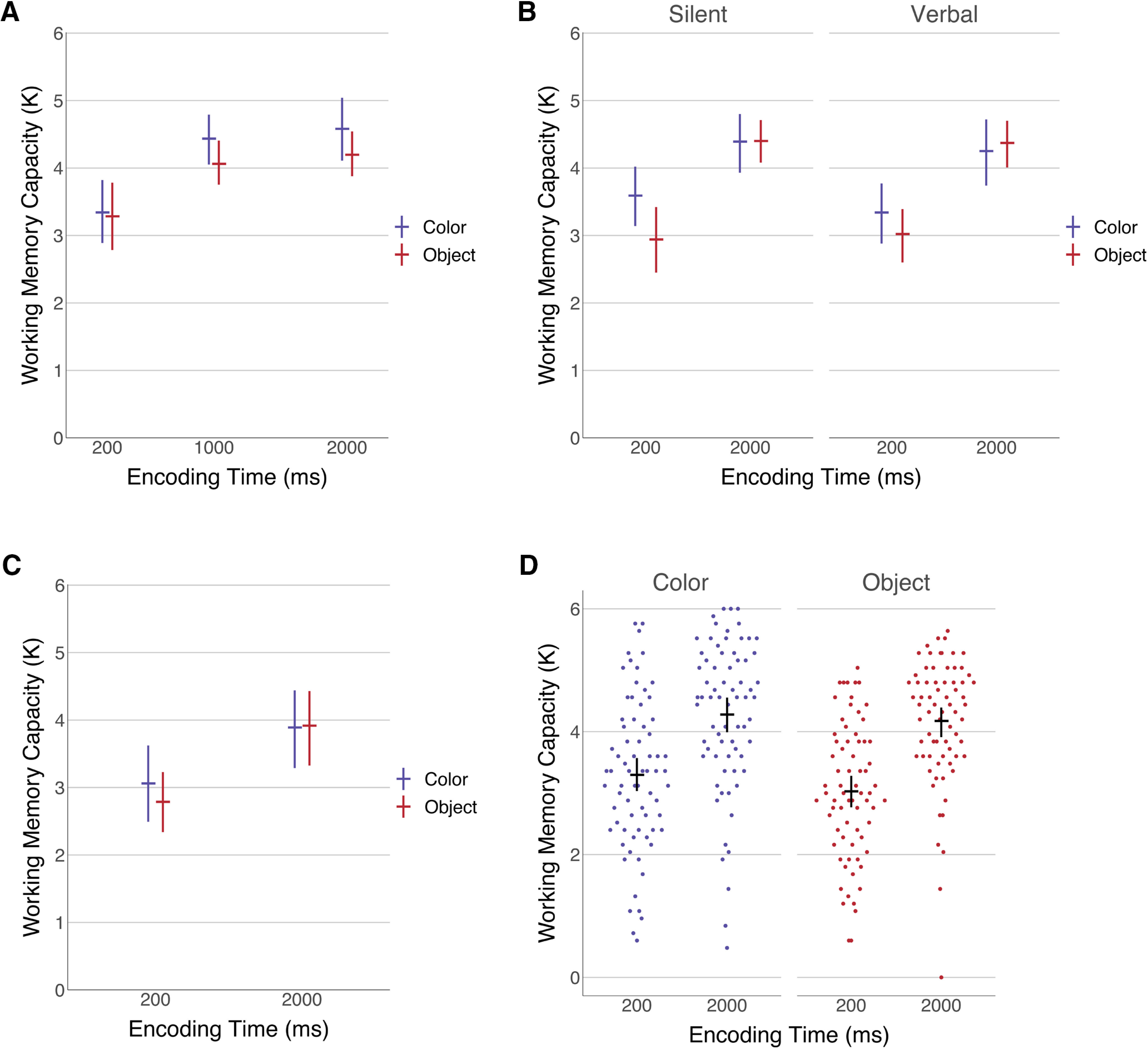
Mean working memory capacity by item type, encoding time, and verbal load. Error bars indicate bootstrapped 95% confidence intervals to illustrate the data independent of modeling decisions such as priors. ***A***, Experiment 1a. A near-direct replication attempt of the critical behavioral findings from Brady et al. (2016). ***B***, Experiment 1b. An additional verbal load manipulation was included in experiment 1b to determine whether additional suppression of verbal strategies impacted results. ***C***, Experiment 1c. A replication of experiment 1a with intermixed trials to further disrupt potential encoding strategies. ***D***, Individual data combined over all three experiments.

We replicated the main encoding time effect for objects observed in experiment 1 from Brady et al. (2016; Fig. 2*A*). At 1000 ms, our model predicted an increase of 0.78 K objects (95% CI [0.37 1.18]) compared with 200 ms. We also observed some evidence for an additional increase when encoding time was extended to 2000 ms, although it was not as conclusive as the increase from 200 to 1000 ms (0.13 K; 95% CI [−0.28 0.55]). For this effect, only 74% of MCMC samples showed a positive difference, compared with 99% for the increase from 200 to 1000 ms. Contrary to the original paper, our results suggested a similar effect of increased performance exists for colors. In fact, the time effect appeared to be even greater for colors than objects, at an estimate of 1.04 K (95% CI [0.63 1.44]). Again, we observed a smaller performance increase as encoding was extended to 2000 ms (0.14 K; 95% CI [−0.28 0.55]). For colors, all 40,000 MCMC samples showed a performance increase from 200 to 1000 ms, but only 75% of samples found a positive effect for the increase to 2000 ms.

Comparing colors and objects at 2000-ms encoding time resulted in no evidence for an object benefit. We observed a slight benefit for colors at the longest encoding times (−0.36 K; 95% CI [−0.79 0.08]), although this result was not fully conclusive with only 94.8% of MCMC samples showing higher color performance. This trend was also present at 1000-ms encoding time, with an observed −0.35 K (95% CI [−0.78 0.08]) performance difference, although no meaningful effect was found at 200 ms (−0.09 K; 95% CI [−0.51 0.34]). This result contrasts sharply with the Brady et al. (2016) results, as it suggests that color performance improved with extra encoding time. Overall accuracy for the two-digit verbal suppression was 95.8% (σ = 3.9), meaning participants did not abandon the rehearsal task.

### Experiment 1b

We were surprised to see that performance for both colors and objects increased with longer encoding times in experiment 1a. In addition to contradicting the Brady and colleagues (2016) result, this result also seemed to contradict previous working memory experiments using simple stimuli finding no difference in performance with encoding time (Vogel et al., 2006; Alvarez and Cavanagh, 2008; Bays and Husain, 2008; Tsubomi et al., 2013). As such, we attempted to rule out any potential differences that could explain this finding. One possible explanation was that the unique blocked design and color testing procedure (i.e., color foil was always 180° away from original) allowed participants to rely on verbal rehearsal strategies. While participants were able to successfully complete the verbal suppression task used in the original study, it was possible that two digits being silently rehearsed was not difficult enough to fully prevent verbal rehearsal strategies (T. Brady, personal communication, Sept. 21, 2016). Experiment 1b was conducted to replicate our own result and to test whether the same pattern would be observed with a larger verbal load.

#### Participants

An additional 25 participants were recruited to participate in experiment 1b. One participant was excluded from the analysis for having a block with below chance performance leading to a final sample of 24 (15 females, 9 males).

#### Stimuli

Stimuli were generated and displayed as described for experiment 1a.

#### Procedure

In experiment 1b, participants were again asked to remember colors or objects, but with an additional manipulation of verbal load. Half of the blocks retained the two-digit silent rehearsal, while the other half involved participants vocally rehearsing four random digits. Microphones were used to record the participants’ speech and were manually checked to ensure the participants were complying with the verbal load instructions. Because the primary effects of interest were the 200- and 2000-ms encoding times, we chose to drop the 1000-ms condition. Participants completed eight total blocks for a total of 400 trials.

#### Analysis

As in experiment 1a, the estimated number of remembered items (K) was calculated for each condition and was used as a response variable for a hierarchical linear model. This model was identical to the first experiment except for the addition of verbal load as an additional interacting predictor. The effect of verbal load was also able to vary over participants. Normal(μ = 0, σ = 1) was used as a prior for the population level verbal load effect and HalfNormal(σ = 1) was used as a prior for the group level effect in line with the logic used in the analysis for experiment 1a. MCMC sampling quality was assessed as described above.

#### Results

The results observed in experiment 1b generally replicated those observed in experiment 1a, we found an overall effect of encoding time, but no difference between objects and colors (Fig. 2*B*). The mean accuracy on the suppression task was 93.7% (σ = 9.6) for the silent condition and 95.6% (σ = 5.9) for the verbal condition, suggesting participants were successful at completing both tasks. We estimated the silent two-digit load condition likely resulted in negligibly higher performance compared with the verbal four-digit blocks (0.08 K; 95% CI [−0.17 0.34]). We then collapsed over verbal load to examine the encoding time effects in aggregate. The results again showed evidence for increased performance with additional encoding time for colors (0.85 K; 95% CI [0.52 1.18]) and for objects (1.39 K; 95% CI [1.05 1.72]). Finally, we attempted to replicate the difference between colors and objects at 2000 ms for each verbal load level. For the silent condition, we expected to replicate our result from experiment 1a showing a small difference in favor of colors. Instead, the results did not indicate a clear difference in either direction with an estimated difference of 0.004 (95% CI [−0.49 0.50]). A similar result was found with the higher verbal load with an observed difference of 0.13 (95% CI [−0.37 0.63]). In both cases, we again failed to replicate the main finding in the original paper showing clearly greater performance for objects compared with colors.

### Experiment 1c

The results of experiment 1b indicated that a larger concurrent verbal load did not mitigate the increased performance for extended viewing times for both colors and objects. However, it is still possible that the improved performance in the long encoding conditions was in part because of the blocked condition design of the original experiment. Specifically, by clustering each factor combination (e.g., object, 2000 ms) into a single block of trials, participants had advance knowledge of the specific condition they would be tested on, which could potentially facilitate idiosyncratic perceptual and mnemonic strategies especially given additional encoding time. We therefore examined the effect of intermixing trials to see whether blocked trials were necessary to observe the encoding time benefit.

#### Participants

Twenty-five additional participants were recruited to participate in experiment 1c. Three were excluded from the analysis because of blocks with performance below chance, leading to a final sample of 22 (14 females, eight males). Because of the limited size of our participant pool, participants from experiments 1a and 1b were allowed to participate in experiment 1c. This resulted in 10 participants in experiment 1c who had participated in experiment 1a or 1b.

#### Stimuli

Stimuli were generated and displayed as described for experiment 1a.

#### Procedure

The procedure largely followed that of experiment 1a. For experiment 1c, trials were not presented in blocks and were instead randomly intermixed. We continued to use only the 200- and 2000-ms encoding time conditions as they were sufficient for exploring the effects of interest. By excluding the 1000-ms level, we were able to present 100 trials per condition allowing us to get highly reliable values for estimated capacity. As verbal load seemed to have no effect, we continued to use the two-digit silent load as used in the original paper.

#### Analysis

The model for experiment 1c was identical to experiment 1a except for the lack of the 1000-ms encoding time level.

#### Results

Here, we again attempted to replicate the encoding time benefit for both colors and objects with intermixed rather than blocked trials, again we observed higher performance at a display time of 2000 ms for both colors and objects with no difference between them (Fig. 2*C*). Performance on the verbal suppression task was again well above chance at 91.6% (σ = 7.4). Despite the intermixed trials, we found a large performance difference between the 2000- and 200-ms encoding time conditions for both colors (0.82 K; 95% CI [0.43 1.21]) and objects (1.12 K; 95% CI [0.72 1.51]). We also tested for a difference between objects and colors at the 2000-ms encoding time and for the third time failed to replicate the primary result from Brady et al. (2016). Instead, we replicated the results from experiment 1b which estimated a negligible difference between the two conditions (0.03 K; 95% CI [−0.38 0.44]).

### Combined experiment 1 results

To generate the best possible estimate of the effect of encoding time and item type based on our data, a final model was created using data across experiments 1a, 1b, and 1c (Fig. 1*D*). The 1000-ms encoding time condition from experiment 1a and four-digit verbal load condition from experiment 1b were dropped to restrict the model to conditions that appeared in all of the experiments. The priors used were consistent with experiment 1a. At the 2000-ms encoding time, we predicted an increase in behavioral performance of 0.94 K (95% CI [0.68 1.21]) for colors and 1.17 K (95% CI [0.90 1.43]) for objects. While we observed better performance for colors than objects at the 200-ms condition (−0.33K; 95% CI [−0.60 −0.06]), the effect was not robust at an encoding time of 2000 ms (−0.11 K; 95% CI [−0.38 0.16]).

We also compared the main effect of performance in the 200- and 2000-ms encoding time conditions across experiments 1a and 1c to determine whether there was a meaningful effect because of blocking trials. Of 40,000 MCMC samples, 93.8% showed a difference between experiments of ∼0.43 K (95% CI [−0.12 0.98]). While this result was not conclusive (especially considering it was conducted between subjects with some overlap in participants unaccounted for by the model), it suggests that blocking conditions may allow for participants to increase their performance.

### Discussion

Across three experiments, we were unable to replicate the primary behavioral result from Brady et al. (2016). Experiment 1a instead showed that memory performance for both colors and objects increased with additional encoding time. As this result conflicted with previous literature finding working memory plateaus after a few hundred milliseconds (Vogel et al., 2006; Alvarez and Cavanagh, 2008), we chose to explore alternative explanations for the performance boost. Experiment 1b replicated the results observed in experiment 1a with an increase in verbal load, arguing against a verbal strategy hypothesis. Experiment 1c further explored the possibility of other nonverbal strategies by disrupting the participants’ knowledge of upcoming trials and, once again, we replicated the pattern seen in experiment 1a. Importantly, no experiments showed evidence that performance for objects was superior to colors at long encoding times. This was a critical component of the argument in Brady et al. (2016) supporting the conclusion that visual working memory capacity is not fixed for real-world objects rich in detail that can be extracted with additional encoding time.

Across the experiments, there are a couple of minor procedural differences that could have resulted in inconsistent findings. In particular, it is possible that the objects with detail condition (which was dropped from our experiments) encouraged participants to perform fine discriminations throughout the rest of the conditions (Allon et al., 2014). If fine discriminations are needed for the critical results to be observed, it could explain why our results fail to match those in the original paper. This is consistent with experiment 3 of Brady et al. (2016), which also dropped this condition leading to smaller effect sizes, although it would not explain why we failed to see any evidence in even the same direction as the original findings with the large number of subjects and trials we recorded.

Based on these results, we see no evidence supporting the strong and specific conclusions in the original paper. Still, we were interested in further examination of the surprising finding that color performance increased with more encoding time. We therefore decided to replicate the final experiment from Brady et al. (2016), which examined differences in CDA amplitude across stimulus types and set sizes.

## Experiment 2

In experiment 3 of the original paper, Brady and colleagues attempt to provide conclusive evidence that the performance benefit they observed for objects versus colors was driven by an increase in working memory capacity. To do this, the authors looked at changes in CDA amplitude, an electrophysiological component known to track the number of items being held in working memory. The CDA is a sustained difference wave (contralateral minus ipsilateral electrodes) observed during the retention period when participants are cued to remember information in either the left or right visual field. CDA amplitude tracks the number of items held in memory up to maximum capacity before reaching an asymptote (i.e., maximum negativity) and strongly correlates with behavior (Unsworth et al., 2015; Luria et al., 2016). Brady et al. suggest that, if working memory capacity is different for colors and objects, it should be reflected in the point at which the CDA asymptotes. Consistent with a capacity difference for objects versus colors, they found that CDA amplitude was comparable for colors and objects at set size 3, but exclusively increased for real-world objects at set size 5. This interaction between set size and stimulus type on CDA amplitude is the critical finding that supports the original authors’ conclusions as a simple main effect of CDA amplitude could be explained by a difference in stimulus complexity (Luria et al., 2010) without supporting a claim regarding an increased capacity per se.

While our procedure closely followed the original design of Brady et al. (2016), we increased the sample size (27 vs 18) and trial counts (240 vs 110 per condition) to improve our expected power to detect any effects, and we monitored fixation with eye trackers rather than relying on electrooculography alone. In addition to the key conditions in the original study, we added a set size 1 condition which would provide a positive control (set size 3 > 1) in the case that we found a null effect for our key comparison of interest (set size 3 vs set size 5). Based on experiment 1, we expected that we would also fail to directly replicate the EEG result reported in Brady et al. (2016; capacity increase for objects but not colors). When we considered our own behavioral effects in light of the Brady et al. (2016) CDA result and the broader literature, we thought two outcomes were most plausible. First, because our results showed a behavioral performance increase for both colors and objects with longer encoding times, we may find that the capacity increase observed by Brady et al. (2016) may actually generalize to all stimuli. If a longer encoding time increases capacity (regardless of stimulus type), then we would predict that the CDA amplitude for set size 5 should be higher for set size three for both objects and colors. Alternatively, the performance benefit that we consistently observed with the 2000-ms encoding time could be because of another confounding task factor (e.g., encoding strategy rather than working memory storage). If so, the observed performance increase is not caused by a true VWM capacity increase and we would expect to observe a typical CDA waveform with no difference between set sizes 3 and 5.

### Participants

A total of 34 participants were recruited to complete experiment 2 for monetary compensation ($15/h). The original paper displayed 110 trials per condition, so we required this number of non-artifact trials in all conditions for all participants. We therefore removed six participants for having too few remaining trials in one or more of the conditions. One additional participant was excluded for having behavioral performance below chance, leading to a final sample of 27 (14 females, 11 males, two other/chose not to respond).

### Stimuli

**Figure 3.**
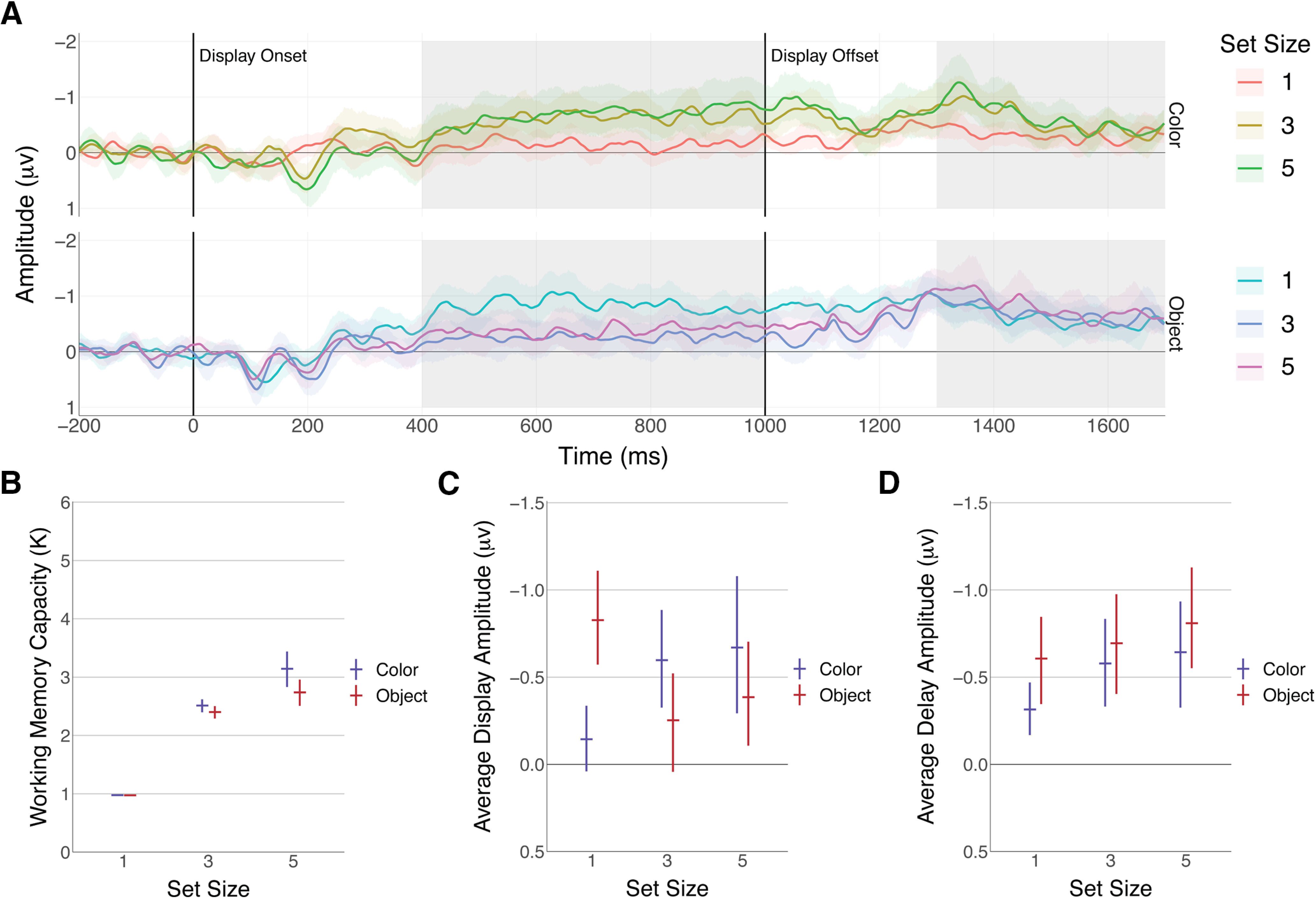
CDA amplitude by set size and item type. Error bars indicate bootstrapped 95% confidence intervals. ***A***, Raw CDA waveforms. CDA was generated by averaging over trials for each participant and calculating a contralateral minus ipsilateral difference wave using the PO3, PO4, PO7, and PO8 electrodes. Highlighted regions show the areas used to calculate mean CDA amplitudes. ***B***, Experiment 2 behavior results. Working memory capacity was calculated using the set size for that condition. ***C***, Mean CDA amplitude for the memory display (400–1000 ms). Means were generated for each participant before bootstrapping. ***D***, Mean CDA amplitude for the delay (1300–1700 ms). Means were calculated using the same time window as Brady et al. (2016).

Color and object stimuli were generated in the same way as experiment 1. In order to account for the lateralized nature of the CDA, five fixed positions (inferred from Fig. 3 in the original paper) were used in each visual hemifield. For set sizes 1 and 3 the same subsets of positions were selected for each trial with nothing displayed in the other locations. The stimuli shown in the more distant positions for set size 5 were approximately M-scaled as described in the original paper (Rovamo et al., 1978).

### Procedure

#### Behavior

As in the other experiments, participants were asked to remember either colors or objects and report which of two choices was the target item. However, as calculating CDA amplitude requires lateralized memory, an arrow displayed at the start of the trial served as a cue directing participants to remember the items in either the left or right visual hemifield. Distractor items were shown in the opposite visual field but were never tested. This arrow remained on screen in place of the fixation cross for the remainder of the trial. Unlike the previous experiments, encoding time was fixed at 1000 ms, a duration shown to result in high behavioral performance. Three different set sizes were tested (1, 3, and 5) and a 700-ms memory delay was used, as this matched the analysis window described in the original paper. No pretrial placeholders or verbal encoding task were used for this experiment. All conditions (two item types × three set sizes) were shown across three blocks of 80 trials for a total of 240 trials per condition (1440 total trials). Blocks were randomly ordered for each participant.

#### Eye tracking

Gaze position was recorded using SR Research Eyelink 1000+ eye trackers at a sampling rate of 1000 Hz. Participants were required to maintain fixation on the cue arrow from the beginning of the memory display until the test was presented. Calibration was repeated before every block to ensure tracking accuracy. While completing the task, participants were instructed to keep their head on a chin rest and avoid moving until their next break.

#### EEG recording and preprocessing

EEG recording was conducted using 32 active Ag/AgCl BrainVision electrodes arranged in a 10/20 system, with two electrodes affixed with stickers to the left and right mastoids. Electrode impedances were lowered to under 10 kΩ during experiment setup and data was sampled at 500 Hz. Horizontal and vertical electrooculograms (HEOG/VEOG) were recorded with pairs of passive electrodes affixed with stickers lateral to the left and right eyes and above and below the right eye, respectively. Scalp recordings were referenced online to the right mastoid and were rereferenced offline to the average of both mastoids. The EEG signal was bandpass filtered online from 0.01 to 80 Hz, then further low-pass filtered to 30 Hz offline. Individual trials were epoched and baselined to the 200 ms before display (Fig. 3*A*).

After preprocessing, an automatic artifact rejection pipeline was applied to reject epochs containing artifacts. A sliding-window step function (window size = 100 ms, step size = 10 ms, threshold = 20 μv) was used on EOG channels to detect eye movements and blinks. A similar sliding window was used for gaze data, with a threshold of 0.1° visual angle, which allowed for tighter control of eye movements compared with using EOG electrodes alone. In cases where artifacts were detected in an EOG channel but not the gaze data, the eye tracking data were preferred. Trials were also rejected for muscle noise if any electrode used in the analysis exceeded a peak to peak threshold of 100 μv. Trials were then manually examined to ensure all true artifacts and eye movements were rejected. After removing participants below the minimum of 110 trials in each condition, participants had a mean of 200 trials per condition (σ = 30). Individual trials were averaged for each participant and a contralateral minus ipsilateral difference wave was created for each condition. The CDA was measured from 1300 to 1700 ms using the average of the PO3, PO4, PO7, and PO8 electrodes. All EEG analyses were done using the EEGLAB and ERPLAB plugins for MATLAB (Delorme and Makeig, 2004; Lopez-Calderon and Luck, 2014). We additionally chose to examine the display window (400–1000 ms), as previous research has shown that the CDA can be observed while items are still in view when using long encoding times (Tsubomi et al., 2013).

### Differences from Brady et al. (2016)

In the original paper, Brady and colleagues describe a matching procedure in which the to-be-remembered stimuli from one trial were used as distractors for another trial. We felt this had no benefit as, after artifact rejection, not all trials would end up matched. We therefore chose not to implement this technique and instead randomly sampled from all possible stimuli for each position individually. We also chose to use a slightly broader bandpass filter on the EEG (25 vs 30 Hz) to avoid accidently filtering out any signal of interest (Luck, 2014). Finally, we used gaze position determined by an eye tracker to determine which trials had artifacts because of eye movements instead of relying on electrooculography as this allowed us to more precisely determine whether subjects fixated the central cross.

### Analysis

#### Behavior

As in experiment 1, accuracy was used to estimate VWM capacity for each condition using the formula from the original paper. Estimated capacity (K) was then used as the response variable in a hierarchical linear model with set size and item type as interacting predictors that varied over participants. The population level intercept, item type, and interaction parameters were all given a prior of Normal(μ = 0, σ = 1) consistent with the logic used for experiment 1a. Because the literature has consistently shown large results related to number of items, set size effects were given the broader prior Normal(μ = 0, σ = 3) at the population and HalfNormal(σ = 3) at the group level. Consistent with the previous analyses, group level parameters were given a HalfNormal(σ = 1) prior with the exception of intercepts, which were given a HalfNormal(μ = 0, σ = 3) prior. LKJ(η **=** 1) was used as a prior for the correlation matrix of group level parameters. MCMC convergence was assessed using the techniques described for experiment 1a.

#### EEG

The model used to analyze the EEG data is identical to the behavior model except the EEG model predicts CDA amplitude instead of estimated capacity. As in the behavior analysis, we expected relatively small effects for all effects other than set size, which is known to result in large CDA amplitude effects. For this reason, the same priors were used in the behavior and EEG models.

### Results

#### Behavior

The behavioral results were consistent with the results observed in experiment 1 (Fig. 3*B*). For colors, the number of remembered items increases from set size 1 to 3 (1.53 K; 95% CI [1.37 1.69]) and from set size 3 to 5 (0.63 K; 95% CI [0.40 0.84]). Objects resulted in a similar increase from 1 to 3 (1.43 K; 95% CI [1.27 1.58]) but showed a smaller increase between 3 and 5 (0.33 K; 95% CI [0.11 0.55]). At set size 5, there did seem to be a modest difference between objects and colors (−0.40 K; 95% CI [−0.55 −0.26]) consistent with the finding in experiment 1a that more colors are remembered than objects. As expected, we again failed to find behavioral evidence of an object benefit.

#### EEG display window

For the display window, we first looked for a typical set size effect (Fig. 3*C*). For colors, CDA amplitude was larger for set size three than for set size one (−0.41 μv; 95% CI [−0.69 −0.14]), but no meaningful difference between set sizes 3 and 5 were observed (−0.07 μv; 95% CI [−0.35 0.20]). This is consistent with multiple previous studies which have found that CDA amplitude reaches an asymptote at working memory capacity (for review, see Luria, et al., 2016). For objects, the results did not a show a typical set size effect. Unusually, CDA amplitude was larger for set size 1 than 3 (0.54 μv; 95% CI [0.26 0.81]), with no clear difference between 3 and 5 (−0.13 μv; 95% CI [−0.41 0.14]). One potential explanation is that CDA amplitude becomes maxed out with a single real-world item leading to overloading often seen when large numbers of items are displayed (Vogel and Machizawa, 2004). To test this, we looked for a difference between colors at set sizes 3 and 5 and objects at set size 1. We found no evidence that the amplitude for colors differed from objects at those set sizes. If anything, it was more likely that objects resulted in a slightly higher amplitude (0.18 μv; 95% CI [−0.12 0.47]).

#### EEG delay period

We then analyzed the time window during the delay period used in the original paper (Fig. 3*D*). The differences between set sizes at this later window were less clear, although there was some evidence for a difference between set sizes 1 and 3 for colors (−0.25 μv; 95% CI [−0.57 0.08]). In total, 93% of MCMC samples showed higher amplitudes for set size 3. The difference between set size 3 and 5, however, showed no effect (−0.06 μv; 95% CI [−0.38 0.24]). For objects, no apparent set size effect was found both between set size 1 and 3 (−0.10 μv; 95% CI [−0.43 0.23]) or between 3 and 5 (−0.11 μv; 95% CI [−0.43 0.20]). We did find a modest effect of item type, showing overall higher CDA amplitudes for objects (−0.19 μv; 95% CI [−0.37 0.00]). Critically, this main effect does not provide support for the original results as there was no reliable evidence of an interaction. The population level parameter describing the interaction between item type and set size three had a value of 0.21 μv_scaled_ (95% CI [−0.33 0.74]) and the coefficient for the interaction between item type and set size 5 had a value of 0.14 μv_scaled_ (95% CI [−0.40 0.66]).

### Discussion

In experiment 2, we attempted to examine the observed encoding time benefit using the CDA. Once again, we were unable to replicate the primary behavioral finding from Brady et al. (2016). In fact, we found the strongest evidence that colors led to higher behavioral performance. This may be because of the fact that we had the highest number of trials and participants in experiment 2, giving us the best chance to observe an effect. Regardless, we found no evidence of any CDA-indexed working memory benefit for real-world objects.

Behavioral performance was much lower than expected based on the results from the first experiment. For colors, we observed a mean K of 3.14 at set size 5 compared with a K of 4.44 for colors at the same encoding duration in experiment 1a. A similar observation was made in the original paper, which was explained as the result of increased task demands and forced fixation in the EEG experiment. We agree that these are plausible explanations, although it is possible that the significant reduction in effect size limits the generalizability of the CDA results to the results from experiment 1a. However, because we did find a performance increase from set size three to set size 5, it is still worthwhile to examine whether this increase is reflected in CDA amplitude.

If the behavior difference for colors and objects at longer encoding times was because of an unusually large VWM capacity, we would expect to observe higher CDA amplitudes for set size 5 consistent with the explanation of Brady et al. (2016). Based on the replication of the analyses from the original paper, we found no compelling evidence that the CDA had a higher asymptote for objects than for colors. Likewise, we found no support that the behavioral performance increase was driven by a true increase in CDA-indexed VWM capacity. Rather, it appears possible that some other confounding task factor, rather than VWM storage, led to a performance benefit with longer encoding times. We also examined CDA amplitude while the sample display was visible, and, once again, failed to find any direct support for the finding that CDA amplitude had a higher maximum for real-world objects.

## General Discussion

The basis of visual working memory has long been debated, although most of the research on the topic has evolved around the idea that individuals have a single maximum capacity (Luck and Vogel, 1997; Wilken and Ma, 2004; Bays and Husain, 2008; Zhang and Luck, 2008; van den Berg et al., 2012; Adam et al., 2017). In contrast to this view, work by Brady et al. (2016) suggests that the capacity of visual working memory is not fixed, but varies as a function of stimulus type and encoding duration. Given the surprising and impactful nature of this finding, our goal was to replicate the key results that led to the conclusion that working memory is not fixed capacity. To this end, we attempted near-direct replications of the behavioral and CDA results reported in the original paper.

In experiments 1a–1c, we observed better behavioral performance with longer encoding times for both colors and objects. Although our results support Brady et al.’s (2016) broad finding that performance on working memory tasks can improve with encoding time, we failed to replicate their key, specific behavioral findings that (1) objects result in overall better performance compared with colors and (2) an encoding time benefit is found for objects but not for colors. The general improvement of performance with encoding time was surprising given previous work, which has generally found that that VWM for simple objects fills within hundreds of milliseconds (Vogel et al., 2006; Alvarez and Cavanagh, 2008; Bays and Husain, 2008; Tsubomi et al., 2013). Fearing that participant strategies unrelated to WM storage were impacting performance, we increased the verbal load (experiment 1b) and intermixed trials (experiment 1c). In all cases, we failed to replicate the original object benefit, and we instead found a general encoding time benefit for both objects and colors. Together, our results suggest an encoding-time dependent performance increase is robust to the disruption of some potential strategies, but further work is needed to understand the factors that cause an encoding time benefit to be present or absent.

Overall, we do not have a clear explanation for why we were unable to observe the key behavioral findings seen in Brady et al. (2016). Our behavioral results combined over 25,000 trials recorded over 71 sessions, so we should have had adequate trial counts to detect the effects observed by the original paper (which had fewer than 17,000 trials over 42 sessions). Although no replication can be perfectly identical by virtue of being conducted at different institutions by different researchers, we attempted to carefully replicate the exact stimuli, timing and key procedures. We believe the few changes we did make were so minor (e.g., leaving out one blocked-condition not relevant to our hypotheses) that if these changes alone fundamentally altered the key result it would severely undermine the importance of the published findings for broad theories of working memory.

While experiment 1 was a conclusive failure to replicate, it raised an unforeseen research question. Why did colors show higher performance at longer encoding times when many previous results suggested they should not? Was increased performance for colors with extended viewing times driven by an increase in working memory delay period activity, as proposed by Brady et al. (2016)? Or, might it be because of a non VWM-task factor such as improved LTM encoding (Shaffer and Shiffrin, 1972; Tversky and Sherman, 1975)? To test whether an increase in CDA amplitude was related to the improvement in behavioral performance for both colors and objects, we ran a replication of the final ERP experiment reported in Brady et al. (2016). In this experiment, CDA amplitude was used as a neural measure of the number of items held in VWM during the retention period. During the time window 1300–1700 ms after stimulus onset, Brady et al. (2016) found an increase in CDA amplitude from set size 3 to 5 only when there was a behavioral improvement from three to five items (i.e., for objects, but not colors). Since we found a behavioral increase from set size 3 to 5 for both colors and objects, based on Brady et al.’s (2016) results, we predicted that we should have found a CDA increase for both colors and objects. However, our results were inconsistent with this prediction, and we failed to replicate Brady et al.’s (2016) finding that a CDA amplitude increase from three to five items co-occurred with the observed behavioral performance increase from three to five items at long encoding times. Instead, we found equivalent CDA amplitude for set size 3 and 5 in both the object and color conditions.

Visual inspection of the waveforms in experiment 2 suggested that the CDA was more robust during the encoding period than during the delay (Tsubomi, et al., 2013). We therefore also quantified CDA amplitude from 400 to 1000 ms after stimulus onset (while items were still visible). During this time period, we found a clear set size effect for color, but no increased asymptote from three to five items. For objects, we found an unusual reverse set size effect, such that set size 1 resulted in the largest CDA amplitude. As the original paper did not include the set size 1 condition, it is difficult to compare this result with theirs. However, inspection of the Brady et al. (2016) waveforms shows that CDA amplitude during the display was much lower for three objects compared with three colors. For set size five, however, there was apparently no difference between colors and objects. It is not clear why objects would show a reverse set size effect during encoding as, behaviorally, we observed higher capacity estimates with larger set sizes. One potential explanation is that participants were less reliant on VWM when asked to remember more than one object and instead used some other strategy (e.g., LTM; verbalization) that was invisible to our ERP analysis. We are not sure why this pattern would be present for objects but not colors, although it is possible that colors (chosen from a fine-grained, continuous range) were simply not amenable to non-visual strategies.

While we were not able to replicate the key behavioral pattern or the main CDA result reported by Brady and colleagues, we did observe some interesting differences between colors and objects in experiment 2. Specifically, we found lower behavioral performance and lower CDA amplitudes for objects than colors, suggesting that the additional complexity of objects may have resulted in fewer items being stored. This finding supports the growing body of evidence complexity may have an impact on the information stored in VWM (Alvarez and Cavanagh, 2004; Hardman and Cowan, 2015). Given the unusual reverse set size effect during encoding of real-world objects, our CDA results also suggest that participants may use a different strategy for remembering familiar objects than colors. One potential hypothesis is that familiar objects can be more readily stored in long-term memory (Xie and Zhang, 2018). Individuals may choose to use long encoding times to offload information from working memory, rather than attempting to store a precise representation in VWM. Future work with real-world items is necessary to further understand the differences between the stimuli used in experiments and VWM processes in real life situations.

Ultimately, we failed to find any direct support for the hypothesis that an increase in working memory storage capacity (as indexed by CDA amplitude) explains an improvement in behavioral performance with longer encoding times. Although we observed a performance benefit with longer encoding times for both colors and objects, we found no direct evidence that this was because of an increase in VWM storage capacity (i.e., we never observed a difference in the CDA between set size 3 and 5). Our conclusions thus stand in stark contrast to those of Brady et al. (2016). However, as our effects are null results, we do not have conclusive evidence toward any particular alternative model. Our goal when designing this work was to initially replicate then later extend the core result, so our experiments were designed with that goal in mind. As a result, it is possible that the very minor procedural difference led to inconsistent results (e.g., omission of the fine-grained object change condition in experiment 1a). Although no replication attempt can ever be truly identical, taken together, a range of replications from near-direct to more conceptual are useful for determining potential constraints on the generalizability of a given result (Schmidt, 2009; Simons et al., 2017). Together, our data suggest that more work is needed by multiple groups to understand the degree to which the results in Brady et al. (2016) are broadly generalizable as suggested in their initial conclusions, versus highly idiosyncratic to the specific sample and/or minor procedural differences.

## Synthesis

Reviewing Editor: Bradley Postle, University of Wisconsin Decisions are customarily a result of the Reviewing Editor and the peer reviewers coming together and discussing their recommendations until a consensus is reached. When revisions are invited, a fact-based synthesis statement explaining their decision and outlining what is needed to prepare a revision will be listed below. The following reviewer(s) agreed to reveal their identity: Stephen Emrich, Jonathan Flombaum.

In the 3-way consultation a consensus emerged from the overlapping observations (in the original reviews, see below) that two factors argue against your stating your conclusions as unequivocally as you do in the current manuscript: First, the (subtle?) differences between your procedures and those of Brady et al. (2016); second, the fact that your behavioral vs. CDA results diverge at some points, and in these cases your tendency is to overlook the former and rely exclusively on the latter to bolster your interpretations. We had some discussion about whether we should request an additional experiment that is a “note-for-note” replication of the Brady et al. (2016) methods. It was mostly hypothetical in tone, and we‘re not going to actually make this request, but I want to be sure to convey the tone of the discussion. The consensus opinion is that, for a revision to be successful, the two points raised above will need to be taken into account explicitly: First, what does it mean that behavior performance IS sensitive to exposure duration (and how does one make sense out of behavior-CDA divergence) ; and second, the strength of your interpretations need to be softened, because clearly many would not agree (including the reviewers!) that your result have definitively resolved this question. I‘ll also share that, in the course of our consultation, it came up that an idealized long-term outcome from this work might be an adversarial collaboration between the two groups in which a definitive design and set of predictions was established a priori and preregistered. But that‘s outside the scope of this manuscript...

Here is the full text of the two reviews.

R1:

The current manuscript describes a series of studies attempting to replicate the effects of Brady, Störmer, & Alvarez (2016): namely, an increased capacity for real-world objects at longer encoding times, with an accompanied increase in CDA amplitude. The authors conducted 3 behavioral studies and 1 EEG study, and in each case failed to replicated the main findings of Brady et al.

Overall I thought this was a well-written paper, and represents an important attempt to replicate a critical finding in the field. A failure to replicate is potentially informative, although I have one major issue concerning some aspects of the design:

Major issues:

1) The critical difference between the current study and that of Brady et al. (2016) is the absence of the inclusion of the “objects with detail” condition from the original study. Although the authors note that this condition was dropped in Exp 3 of the original study, that experiment also produced a much smaller difference between objects and colors than the first experiment of the original study. Thus, rather than a pure failure to replicate, this finding may just support the possibility that fine discriminations are required between real world objects in order to observe robust effects.

This is particularly important as there is evidence for similar effects in the literature. For example, Allon et al. (2014) demonstrated that when having to distinguish between two polygons from different sets (large changes) the CDA for one polygon was much smaller compared to when having to make distinctions between two polygons from the same set (small changes). Although there are a number of differences between that design and the current study ( as well as observing a potential asymptote at higher set sizes, consistent with the authors‘ effects), it nevertheless reveals that it‘s at least possible that the objects with detail condition could be critical to producing an increase in the CDA amplitude at longer encoding durations. Thus while the authors go to great lengths to carefully rule out possible mechanisms (verbal encoding load, blocked vs. random trials), it seems to me that this remains a potential factor that should be ruled out. And although I agree with the authors that “if these changes alone fundamentally altered the key result it would severely undermine the importance of the published findings for broad theories of working memory.” (p 26), it‘s also possible that this condition could put boundary conditions on exactly what the mechanisms are that do or do not produce this effect, which could be an even more useful contribution to the field.

Other issues:

2) I wonder whether the lack of an observed effect could be attributed to the relatively small overall CDA amplitudes. The color condition has a max amplitude of less than a microvolt, which is quite a bit smaller than is typical. Even in comparison to the original Brady et al (2016) paper the amplitudes are quite a bit smaller. I suspect the authors would argue that if the effect depends on, for example, the spatial configuration of the stimuli, then it limits its importance (which I would agree with); however, it does make me wonder if there is something methodologically that is limiting the CDA signal, which could mitigate any potential effects.

3) Relatedly, I think it would be useful to provide more details about eye-movements rejection criteria, and whether it is possible to identify residual eye-movements (were HEOG recorded at all?) as this could affect the CDA amplitude.

4) Are statistical analyses performed on the second band-pass filter of 0.1-30 Hz? If so, this is somewhat more aggressive than what I think is typically recommended (e.g., Luck text, 2014). This is of particular relevance here as even a high-pass of 0.1 can attenuate the effect of late slow-wave components like the CDA (Tanner et al., 2015).

5) The authors state that “Based on our experience and the effect sizes reported in the literature, we believe that it is unlikely to observe a scaled effect greater than 2 standard deviations,” (p9). Is it possible that an SD greater than 2 would change the results meaningfully? Particularly if effects for real-world objects show greater variance?

6) I don‘t agree with the conclusion that “Thus, we found no support for increased VWM capacity with either stimulus type.” (p 27), which seems to be based on the CDA results alone, and ignores what appears to be a pretty robust behavioural effect across experiments (e.g., Figure 2D)

References:

Allon, A. S., Balaban, H., & Luria, R. (2014). How low can you go? Changing the resolution of novel complex objects in visual working memory according to task demands. Frontiers in psychology, 5, 265.

Tanner, D., Morgan‐Short, K., & Luck, S. J. (2015). How inappropriate high‐pass filters can produce artifactual effects and incorrect conclusions in ERP studies of language and cognition. Psychophysiology, 52(8), 997-1009.

R2

Apologies for the delay submitting this review. In an effort to keep this short, I‘ll summarize what they did simply: They re-conducted some of the experiments from Brady et al. not failing to find effects, but failing to find the same effects (and thereby finding others). Importantly, these authors find better WM performance with increasing viewing time for both real world objects and colors, whereas Brady et al. only found it for RW objects. And they did not find a difference in the CDA for RW objects vs. colors regardless of load.

With respect to the impact and appropriateness for the journal, I need to leave that to the editor and other reviewers. This is a well done study, and the Brady paper is a high profile and important result. So arguably this is too. I certainly welcome and value this report, and its not really a failure to replicate. On the other hand, what‘s new are the results not the experiment. It seems to me that ultimately this paper raises more questions than it could answer: whose results should we trust? I hope two or more other groups will replicate this.

All that said, I only have one methodological suggestion, a small interpretive concern, and then a sense of confusion about the topic that applies equally to this paper as to the original one by Brady.

Methods suggestion: For experiment 2, the authors note a number of things they did differently, because they seemed either minor or unneeded. Lines 443-452. E.g. they did not use the matching procedure or pick foils in the same way as Brady. I agree with the authors; it seems really unlikely that these differences caused the different experimental outcomes.... But given the stakes, maybe its worth trying. It is possible for example that the over the course of the experiment the foil choices change the subjects‘ perception of difficulty in the experiment, even their overall confidence, in some way that affects the CDA. Anyway, this is the one spot where I could imagine one more experiment being useful, and beneficial ultimately even as just one more attempt to tilt the scales in terms of which results to trust. [I assume that the authors don‘t know why the Brady group got what they got, and so with respect to Experiment 2 we basically have 2 near identical runs of the experiment, one producing an effect that the other does not. I appreciate that the authors trust their results. But for the rest of us it is hardly definitive].

Relatedly, why do the authors seem to think that the CDA effects are correct though they are incompatible with the presence of an effect in the behavioral experiment. I.e. why does exposure time affect behavioral performance, but not the CDA, and why doe that mean that capacity is not changing as opposed to the CDA not always reflecting all capacity changes?

My main feeling after reading this paper is that the relationship between theory, measurement, and psychology is muddled in ways that the field has perhaps not appreciated or at at least emphasized. A few thoughts that apply to the Brady paper as well -so not really a criticism of this paper per se. Just things that make it challenging for me to find meaning in the results regardless of how they are as a matter of fact.

1. Something is not right with using K as the measure of behavior in order to study effects of exposure time. The K measure assumes a discrete capacity limit. It is designed to provide a value, K, of what that limit is. That means that it can only show changes to discrete limits. So if exposure time has an effect that is not to increase (or decrease) a discrete limit it will always look like that sort of effect if measured by K. Note that in the intro the authors say “Importantly, this behavioral result alone does not distinguish between a general performance benefit and a true increase in working memory capacity (i.e., a higher limit on the amount of information stored).” A good point. But I think one that applies to their own results as well.

2. There is just something not right about the conception of working memory capacity. I think the issue is that the authors (and their opponents) flip freely between a view of working memory as limited by a limited substance vs. a view in which the limitations are a property that emerges from the nature the dynamics in the underlying substrate. If WM limits are caused by a substance, then how could they increase for this category or that one in the first place? The whole notion does not make sense to me. If I have one bar of chocolate, the number of grams won‘t change if I decide to distribute it to colored squares or RW objects. For example, they characterize Brady as follows: “This was a critical component of the argument in Brady, Störmer, Alvarez (2016) supporting the conclusion that visual working memory capacity is not fixed for real-world objects rich in detail that can be extracted with additional encoding time.” But with a substance view of VWM, Brady‘s position is incoherent to begin with. How could I have more capacity because I am storing RW objects? I think that what is going on here is not that Brady et al. think that WM is not substance-limited, but effectively limited, i.e. that the limits are a property of the system that emerge from the dynamics. I think the authors of this paper believe WM is actually limited by the size of the vehicle that stores the information. If this is so, it would be much better to couch the disagreement, and the interpretation of the results in these ways. If I am wrong, then it is worth being explicit about what sort of limit is being tested, and then one needs to make sure that the results and theories make sense in relation to that kind of limit.

3. “Brady, Störmer & Alvarez suggest that, if working memory capacity is different for  

colors and objects, it should be reflected in the point at which the CDA asymptotes.” (line 364). True, but I think the authors agree with this, and use it to support their argument because they get different results. My concern is that both teams are accepting a premise that seems questionable: that the CDA always and only reflects discrete capacity, a situation in which both the presence and absence of evidence each can and always do mean one specific thing. See also line 611-615
